# A New Conceptual Framework for Teacher Identity Development

**DOI:** 10.3389/fpsyg.2022.876395

**Published:** 2022-05-09

**Authors:** Reza Pishghadam, Jawad Golzar, Mir Abdullah Miri

**Affiliations:** ^1^Department of English, Ferdowsi University of Mashhad, Mashhad, Iran; ^2^Department of English, Herat University, Herat, Afghanistan

**Keywords:** identity, capital, emotioncy, investment, imagination of reality, discourse, mirrors of power

## Abstract

Teacher identity has evolved from a core, inner, fixed, linear construct to a dynamic, multifaceted, context-dependent, dialogical, and intrinsically related phenomenon. Since little research has provided an inclusive framework to study teacher identity construction, this article proposes a novel conceptual framework that includes the following components: mirrors of power, discourse, the imagination of reality, investment, emotioncy, and capital. The above core constituents have been discussed thoroughly to trigger significant insights about teacher identity development.

## Introduction

A burgeoning body of research on teacher identity has gained momentum in education, emphasizing teachers’ learning, performance, and cognition (Beauchamp and Thomas, 2009; Miller, 2009; Akkerman and Meijer, 2011). In view of recent studies, teacher identity has been conceptualized in different ways. Most scholars emphasized the teacher identity as dynamic, evolving, multiple, and pertinent to intrinsic interpersonal relationships with others (Leigh, 2019; Golzar, 2020). Some others employed a dialogical approach to look at identity as influential to the teachers’ mentality and practices (Akkerman and Meijer, 2011). Some scholars emphasized the place of emotion in a richer understanding of identity development (Zembylas, 2003; Shapiro, 2010; Schutz and Lee, 2014; Kocabaş-Gedik and Ortaçtepe Hart, 2021). Song (2016) argued that the emotional responses allow us to trace the subjectivity of language teachers to its institutional and social contexts.

Several factors contribute to developing teacher identity in the classroom, including demographical indexes (age, gender, and education); sociocultural, economic, and institutional dynamics (Duff and Uchida, 1997; Lin et al., 2002; Olsen, 2008; Danielewicz, 2014; Li, 2020), as well as discriminatory behavior toward non-native English speakers inside the inner circle or beyond and marginalizing them based on the existing binary (Kachru, 1986; Jenkins, 2006). Furthermore, both culture and teacher identity play a significant role in shaping effective teaching practices, which can be best explained through the emotional and social dynamics of the classroom (Good et al., 2009). Perceiving the implications of teacher identity is pivotal for improving and sustaining teaching standards (Day et al., 2006; Kayi-Aydar, 2015; Barger, 2022). In addition, “professional competence came to the fore being the best mediator to gain awareness of professional teacher identity” (Richards, 2021, p. 204). Understanding different aspects of teacher identity can be employed as a framework through which researchers can investigate teaching processes and methods to incorporate skills into many relevant identity tensions in the workplace (Olsen, 2008).

Considering its significant effects on teacher education, many researchers employed different methods to study identity formation in terms of discourses the teachers yield and get involved in Alsup (2006); of effects of the contextual factors (Van Lankveld et al., 2017), and of narratives which the teachers construct to describe their inner and professional worlds (Park, 2012; Li, 2020). The selection of a theoretical framework for examining teacher identity depends on several factors, including inherent sociocultural processes, the researchers’ beliefs, the complexity of the construct, use of various research methods, and further insights into that particular framework (Ahmad et al., 2019). Some scholars proposed different frameworks for investigating language teacher identity development (Kaplan et al., 2015; Trent, 2016; Yazan, 2018). Trent (2016) maintained a poststructuralist view toward identity—integrated time and space as latent sites of incongruity and conflicts; emphasized the role of discourse to understand an individual’s self, identity, and agency. Kaplan et al.’s (2015) teacher identity framework was comprised of four unique yet interrelated constituents: (1) epistemological and ontological views; (2) aims and goals; (3) self-image; and (4) perceived possibilities of practice. Yazan (2018) proposed a multidimensional framework consisting of teacher’s learning, cognition, participation in communities of practice, relevant contextual factors, biographies, and emotions. However, teacher identity appears to be demanding to fully represent. It is “a complex mélange of influences and effects in which macro- and microsocial histories, contexts, and positionings combine with the uniqueness of any person to create a situated, ever-developing self that both guides and results from experience” (Olsen, 2011, p. 259). In this regard, a single theory is not enough to conceptualize this complex and multifaceted construct. Moreover, examining relevant contributing factors and constituents has been given a short shrift by research scholars. The holistic understanding of teacher identity development requires proposing a more inclusive and well-grounded conceptual framework (Zembylas, 2018; Ahmad et al., 2019).

**Figure 1:**
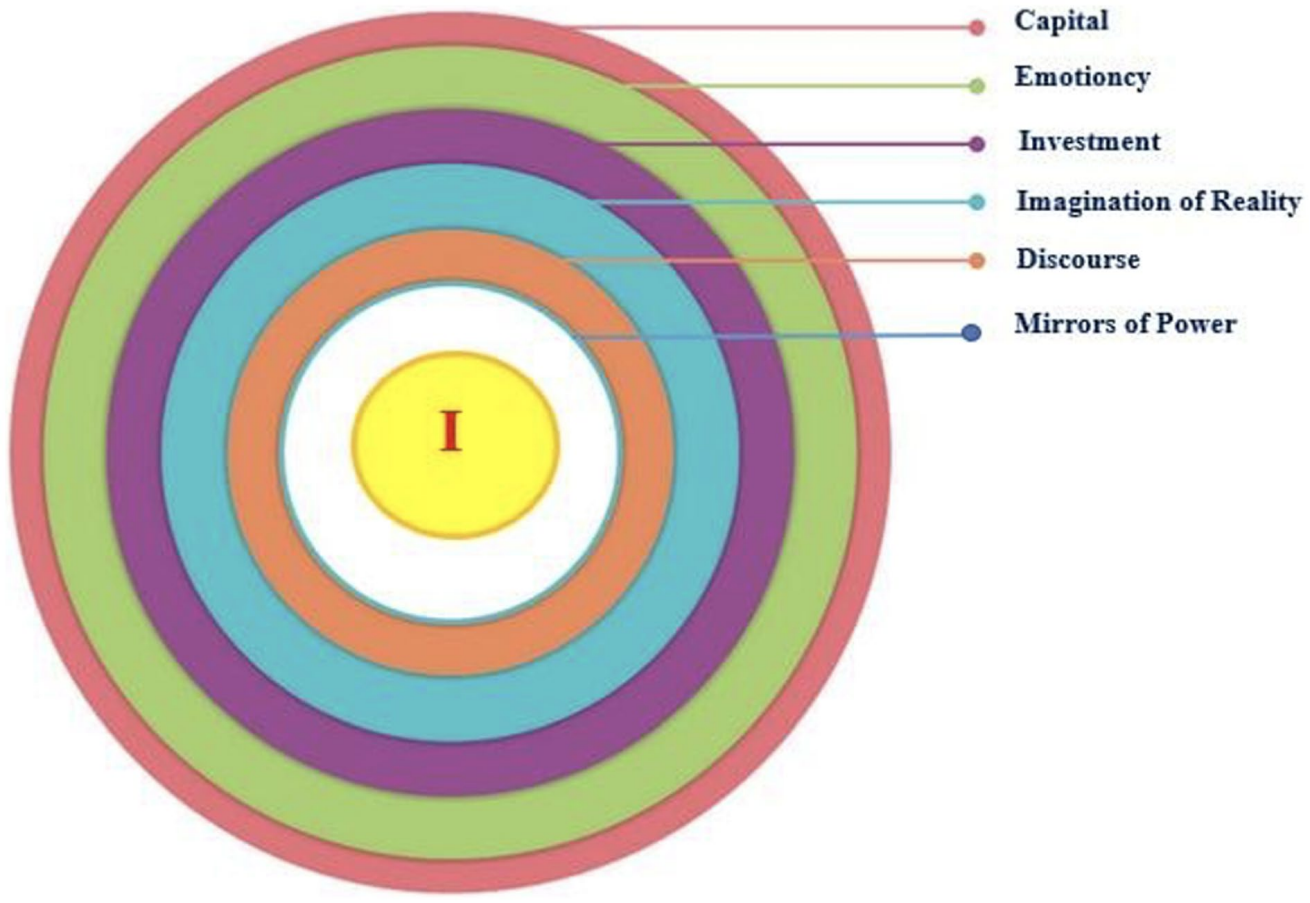
A new conceptual framework for teacher identity.

This paper aims to conceptualize teacher identity pursuing a more comprehensive approach by reviewing existing scholarship. We also trace identity through psychological as well as sociological perspectives. Ultimately, we introduce a new framework to obtain a more holistic understanding of teaching identity dimensions while developing a transformative perspective toward sense-induced emotions and its cognitive load on identity formation. This framework includes core constituents, such as (a) *mirrors of power*, (b) *discourse*, (c) *imagination of reality*, (d) *investment*, and (e) *emotioncy*, and (f) *capital* (see Figure 1).

## Conceptualizing Identity

Identity is a concept that has been studied in various ways within the bulk of research. In the essentialist view, identity is a set of personal peculiarities exclusively connected to a specific person. It entails an inner core that appears in childhood and comes to the surface as life goes on, yet it does not change (Hall and Du Gay, 1996). This core identity includes personal attributes and characteristics of the individual (Jones and McEwen, 2000). These attributes are self-descriptive and associated with identity categories that determine the self (Deaux, 1993). According to Waterman (1985), identity refers to “having a clearly delineated self-definition, a self-definition comprised of those goals, values, and beliefs which the person finds personally expressive, and to which he or she is unequivocally committed” (p. 6). In constructive view, however, *self* exists in two spaces simultaneously: I and other; they have a semiotic relationship and work constantly to shape the self (Bakhtin, 1990). Identity should be conceptualized as an image of self-living and developing through social activities (Holland et al., 1998; Pishghadam et al., 2013b). Therefore, essentialist frameworks consider identity as measurable, fixed, linear, and predetermined within classifications, such as gender or ethnicity. However, constructivist models viewed identity as fluid and constructed within a complex network of social interactions (Norton, 1997).

Structuralism was coined and advanced by various prominent scholars, such as de Saussure, Lévi-Strauss, Lacan, and Foucault, in the late 20th century. It is an intellectual approach that aims to examine a specific area as a complex system, comprised of interconnected constituents. It is also interested in the relationship between these components and “where objects are defined by the set of relationships of which they are part and not by the qualities possessed by them taken in isolation” (Barbosa de Almeida, 2015, p. 626). It pursues to cognize and clarify social reality in the sense of social structures (Heydebrand, 2001). Structuralism, therefore, holds a view in which all human activities, including behaviors, perceptions, and beliefs, are constructed and not natural. However, it has been criticized for its unwarranted rigidity and its emphasis on deterministic structural forces regardless of the individual subject’s aptitude to perform his role in the context. Challenging the tenets of structuralism, poststructuralism was developed as an intellectual approach to criticize the structuralist binary oppositions and rigid nature. It considers identity as a continuous process and contested construct of positioning, discourses, and power relations (Zembylas, 2003). See Table 1 for a summary of identity theories.

**Table 1:** Understanding identity development through evolving theories.

**Theories**	**Core values**
Essentialism	It views identity as static, measurable, linear, and programmed within different categorical indexes, including ethnicity, gender (Medin, 1989; Norton Peirce, 1995).
Constructivism	It views identity as dynamic and formed within an intricate web of social relations (Bakhtin, 1990; Norton, 1998).
Structuralism	It looks at identity as a complex system with interrelated components and seeks to understand their relationships (Barbosa de Almeida, 2015).
Poststructuralism	The poststructural identity is “multiple presentations of self which are (re)constructed across social contexts and demonstrated through actions and emotions, [it is] multifaceted, dynamic, a site of struggle, and shaped by power relations between the individual and others” (Kayi-Aydar, 2015, p. 138).

With the emphasis on social influence, identity is considered a construct positioned in mind and within a social context (Heisey, 2011). Gee’s (1999) conceptualized identity as enacted through multiple ways of thinking, acting, and interacting so that it became recognized as a certain kind of person. It is also developing in a process and is context-specific, shifting from context to context (Hall, 2000). People negotiate their identities depending on whom they interact with and the environment in which these interactions take place and identity is relational. For instance, the role of a teacher depends on the role of their students or their supervisors (Carter and Mireles, 2015).

Moreover, identity is multifaceted, plural (Cummins, 2011), and “dynamic rather than stable, a constantly evolving phenomenon” (Beauchamp and Thomas, 2009, p. 177), and it is also bound to multiple internal and external factors (Yazan, 2018; Fairley, 2020). Identity also plays a pivotal role in developing teachers’ professional learning (Buchanan, 2015). Therefore, identity functions as a bridging concept that facilitates the study of individual learning, socially situated experiences, and their relationship (Farnsworth, 2010).

## Identity and Psychosociology

Many scholars have examined identity development through psychological perspectives. Erikson (1968) also established and introduced a model for psychosocial stages of identity development. A person’s identity is developed through a continuous process through which the individual explores different choices in his/her lifespan and then makes a commitment toward each particular decision. Performing such adult tasks requires individuals to create a shared synergic relationship with the community and sustain a sense of continuity within the self. If a person fails in his/her commitment, identity diffusion occurs as a polar outcome of this psychological crisis (Erikson, 1963). Erikson’s theory of psychosocial development also delineated eight stages from infancy to adulthood. A crisis exists in each stage that an individual is required to handle. If the person successfully performs during a particular stage, it enhances individual competence. In other words, mastery leads to ego strength. If not, it creates a sense of shortfall within the self. Erikson (1968) employed the term crisis “in a developmental sense to connote not a threat of catastrophe, but a turning point, a crucial period of increased vulnerability and heightened potential” (p. 96). Identity versus role confusion is one of the crises that occurs in adolescence. Role confusion creates a variety of experiences in the individuals. A person may interrogate one of his or her main personal peculiarities, his perceptions of self as well as the surrounding world (Bosma et al., 1994). Such experiential events cast doubt on his existence and role in the context in which he interacts with others.

Building upon Erikson’s theory of identity, Marcia (1993) argued that identity development starts with a combination of beliefs, values, identity indexes, and childhood skills that offer persistent cohesion with past experiences and delineate routes for the future. Marcia (1966) categorized four major positions within the identity formation process: identity *diffusion*, identity *foreclosure*, *moratorium*, and *identity achievement*. He believed that people experience a series of crises or turning points which function as catalysts provoking changes, internal encounters, and emotional turmoil within the process that, in turn, lead to questioning one’s value system. The aftermath of these crises shapes a specific identity by exploring a variety of possibilities to establish new beliefs and make various decisions. Opposite of Erikson’s theory, Marcia argued that an adult could experience his identity status move in several directions. For example, suppose a teacher experiences emotional havoc during his career. In that case, the event can result in a reappraisal of previously held beliefs, either going back to the former position or upgrading to a new one as he adopts new values. Inspired by Marcia’s (1966) identity statuses, Sayah et al. (2014) validated the identity scale by running Structural Equation Modeling (SEM). They examined the participants’ age, gender, and school differences across two contexts (English institutes and public schools). Through Principle Component Analysis, they found four interrelated factors: religious identity, social interpersonal identity, cultural identity, and political identity. Sayah et al. (2014) also found that the participants obtained a higher score in perceiving the four statues in English institutes compared to public schools. Moreover, the study revealed that English institute’s students were moratorium and identity achievers at social interpersonal construct compared to public schools. It entailed a delocalization notion in which participants’ natural tendency toward a foreign culture situates them within identity change unswervingly.

In this regard, Waterman (1985, p. 57) argued, “adolescents differ with respect to the number of identity elements they consider within any particular domain, the vigor with which they explore the limits of each and degree of personal investment they feel in the options eventually adopted.” Emphasizing the role of society and context on identity development, Erikson (1959) argued that the relationship between an individual and society is convolutely interwoven and vigorously associated with a constant shift.

## A New Conceptual Framework for Teacher Identity

A conceptual framework is a structure that researchers employ to explain the development of a phenomenon (Camp, 2001). It helps the researchers recognize and clarify what they know, be concerned about, and respect the main facets of research and ultimately tie them with other constructs (Ravitch and Riggan, 2016). A conceptual framework also maps out the main dynamics of a study, variables, and features and then postulates the connections among those aspects (Miles and Huberman, 1994). Considering the above functionality, the remainder of this article depicts and discusses a conceptual framework for better understanding teacher identity development (see Figure 1). The framework includes the following components: (a) *mirrors of power*, (b) *discourse*, (c) *imagination of reality*, (d) *investment*, (e) *emotioncy*, and (f) *capital*.

### Mirrors of Power

Pines (1987) argued that mirroring plays a significant role in developing individuals’ coherence and organization, resulting in self-awareness and understanding the relationships between self and world. Mirroring begins in the early stages of child development. An infant learns social skills from their caretakers or parents by mirroring them. Mirroring a social phenomenon can support the child in responding to their parents’ behaviors, building social relationships with others he is interacting with, and seeking comfort (Meltzoff, 1990; Stack and Poulin-Dubois, 2002). Maintaining a social neuroscience perspective, many scholars believe that self is used to simulate another person (Aron et al., 2004; Beer, 2012). According to embodied simulation theory, similar neural structures which are engaged in processing actions and feelings are also active when similar actions and feelings are to be identified in other individuals (Gallese, 2003). Individuals tend to imitate the people or entities whom a higher status and power inhered to their identities (Mintz, 1985). For example, parents, teachers, authorities, or cultures innately possess and present a higher degree of power. They function as mirrors of powers that create a variety of discourses.

### Discourse

Discourse is one of the notions that influences identity development (Miller, 2009), and it has a subject for scholarly discussions focusing on social theory. It is also a multifaceted and broadly perceived concept (Pitsoe and Letseka, 2013). In the light of poststructuralism, Foucault (1982) considered the discourse as various ways of knowledge making with social practices, power relations, and biases that are naturally attached to these domains and their complex relationships. Language, for instance, manages and adapts the social worlds in a particular way and informs these social practices constructing specific forms of subjectivity. In a similar vein, Clegg (1989) argued that our sense of ourselves as distinct subjectivities is formed through language. Therefore, the discourses tend to govern the subjects by influencing and constituting the ways they think and feel (Weedon, 1987).

In Foucault’s perspective, knowledge is associated with power convolutedly and power is everywhere. Power is understood to constitute and govern the subjects in different knowledge domains and discourses (Brown, 2006), a dynamic system of control or absence of, such as a dominance between the subject and discourses (Weedon, 1987). Power relations and its variety in operation “categorize individuals, marks him by his own individuality, attach him to his own identity, impose a law of truth on him, which he must recognize, and others must recognize in him” that eventually leads to identify formation (Foucault, 1982, p. 212). In this respect, power relations can also be a product of master narratives which are “culturally shared stories that guide thoughts, beliefs, values, and behaviors” (McLean and Syed, 2015, p. 323). Master narratives establish significant restrictions on one’s agency to develop his identity. The individuals who do not conform to the master narratives may have been marginalized in one way or another (Syed et al., 2018). For instance, the dominant narratives could give an inferior validity to different users of English language and create a dichotomy between native and non-native speakers (Pennycook, 1989). Several studies attempted to examine and problematize the NEST-NNEST mutually exclusive difference in teachers’ identity formation (Faez, 2011; Trent, 2016). Faez (2011) argued that the dichotomies between native and non-native English speakers fail to open up opportunities for “the multifaceted linguistic identities of individuals to be revealed”. In a similar vein, Trent (2016, p. 246) found that multiple discourses act reciprocally to facilitate or limit scope and possibilities for teacher identity development.

Identity bears changes in particular discourses. Scollon (1997) argued that individuals are expected to be persons. They present themselves to be in any discourse. Moreover, successful communication between interlocutors depends on how the individuals correspond well to discourse identity and one’s anticipated social identity. Gergen (1994, p. 129) highlighted the effects of social dependence and meaningful discourse on identity development. “The locus of knowledge is no longer taken to be the individual mind but rather the pattern of social relatedness”. As individuals participate in various discourse communities, these multiple memberships divide the self into a multidimensional, dynamic, and context-dependent construct. They reiterate socio-constructivist theory in which reality does not exist in isolation as a free-standing entity, but it is socially formed (De Fina et al., 2006).

### Imagination of Reality

Based on the discourses, a person prescribes his own imagination of reality. Reality is not as objective as it seems, and our perception of reality depends on our sensory system. People may have different interpretations of reality. As a result, hyperreality came to existence as an inability of cognizance to differentiate reality from its simulation. It is a representation of “models of the real” without original accounts. In modern society, media presents history by reporting accounts of the depictions itself and these accumulated accounts to change the reality into hyperreality (Baudrillard, 1994).

That being the case, individuals imagine and perceive a membership in a particular socially constructed community as they are inextricably engaged in the discourses mostly produced by mass media (Anderson, 2006). Membership categories “lock discourses into place, and are therefore ready for opening to critical examination” (Baker, 2000, p. 99). Through imagination, individuals can develop and negotiate their identities and modify their own realities. To create a sense of belonging to the community, the members had to embrace different perspectives and meanings and attach them to their identities (Wenger, 1998). Kanno and Norton (2003) argued that imagined communities are not immediately concrete and reachable.

Wenger (1998) defined community of practice (CoP) as self-governing system which can be studied considering the three perspectives: what the community is about, how it functions, and what kind of competence it produces for its members. Wenger (1998, p. 176) believed that imagination is “a process of expanding oneself by transcending out time and space and creating new images of the world and ourselves”. He also argued that learners’ relationship to communities of practice engages both participatory and non-participatory behaviors and that both patterns influence the participants’ multiple identity developments. Therefore, the communities play a significant role in developing identity by informing certain ways of thinking, behaving, and performing (Lave and Wenger, 1991). In the light of imagined community and CoP, identity is “an ongoing process of negotiating and interrelating multiple I positions in such a way that a more or less coherent and consistent sense of self is maintained throughout various participations and self-investments in one’s (working) life” (Akkerman and Meijer, 2011, p. 8). Bowen et al. (2021) argued that teachers can legitimize well-liked positions through investments which replicate the degree of their agency in the field.

### Investment

Another important construct in our identity model is investment, which is affected by the unequal relations of power individuals experience as a result of various contextual mandates (Norton Peirce, 1995, 2013). Norton Peirce’s (1995, 2013) poststructural orientation to construct of investment was informed by Bourdieu’s (1992) notions of capital and symbolic power. She argued that language learners usually invest in L2, aiming to acquire more symbolic resources (e.g., education) and material resources (e.g., money), resulting in increasing their social power and cultural capital (Norton, 2013). Her construct of investment, unlike motivation, conceives learners as individuals with dynamic and complex identity, which changes across time and space based on the individual’s interaction in various contexts. Borrowing Norton’s construct of investment, in our model, we postulate that a teacher’s identity, which is considered complex, dynamic and evolving, is constructed through the imposition of investment. Investment in this sense is a matter of action. For example, when a teacher decides to do something, it means the teacher has decided to act. Thus, the action and experience of the teacher affect the way the teacher understands and views the world. This decision to invest and take action is related to the concept of emotioncy since various emotioncy levels (i.e., avolvement, exvolvement, and involvement) can be engaged when having exposure to different items. Ultimately, it reiterates the fact that emotions are inextricably coupled with cognition as Barcelos (2015) argued that the more substantial a belief and more attached to the emotions, the more significant it is to individuals’ identities.

### Emotioncy

Considering the importance of emotions and senses in education, Pishghadam et al. (2013a) asserted that people hold different levels of emotions, induced by senses, toward various items. They named the concept emotioncy (emotion + frequency), a process in which individuals’ “idiosyncratic understanding of the world through their senses” is measured (Pishghadam et al., 2016, p. 4). They added that the construct of emotioncy integrates sense, emotion, and cognition, and the sensory experiences could result in emotions reactions.

Senses and emotions are considered to be intertwined because cognition can be relativized by sense-induced emotions (Pishghadam et al., 2013a). In this respect, due to the notable role of senses in connecting people to their outside world, emotioncy (emotion + frequency) can be used to describe the relationships of senses and emotions. The concept of emotioncy has received much attention in various disciplines, particularly language teaching, neuroscience, psychosociology, and translation (Miri and Pishghadam, 2021). Pishghadam (2016) offered a detailed description of each emotioncy type and emotioncy kind, which is presented in Table 2.

**Table 2:** Emotioncy types and kinds.

Type	Kind	Experience
Avolvement	Null emotioncy	When an individual has not heard about, seen, or experienced an object or a concept
Exvolvement	Audio emotioncy	When an individual has merely heard about a word/concept
	Visual emotioncy	When an individual has both heard about and seen the item
	Kinesthetic emotioncy	When an individual has touched, worked, or played with the real object
Involvement	Inner emotioncy	When an individual has directly experienced the word/concept
	Arch emotioncy	When an individual has done research to get additional information

**Figure 2:**
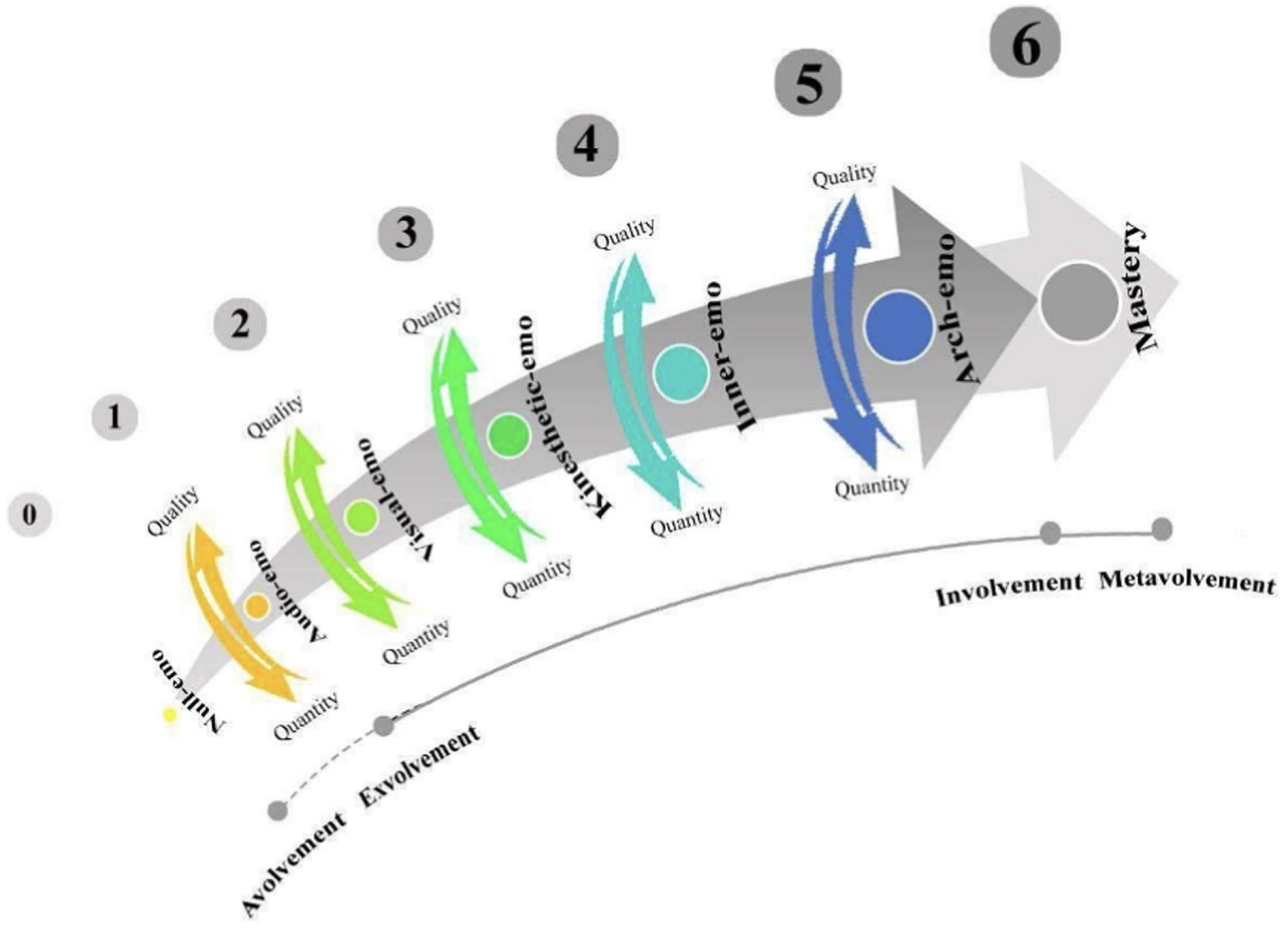
Emotioncy model.

The emotioncy notion has been devised by Pishghadam et al. (2019) into a seven-level matric to demonstrate an individual’s emotioncy level toward a specific concept or phenomenon (see Figure 2).

As depicted in Figure 2, the first emotioncy level is avolvement (null emotioncy), in which the person has no background or experience of the concept. In the exvolvement level, audio emotioncy, visual emotioncy, and kinesthetic emotioncy are involved. However, because each level of emotioncy in the model adds to its previous level, the involvement level (inner emotioncy and arch emotioncy) includes the avolvement and exvolvement levels. The metavolvement level is the ultimate level in the emotioncy matric in which the person reaches the mastery level. In other words, in metavolvement level, the person produces the content that can be used (i.e., heard, seen, and touched) in exvolvement and involvement levels. Due to the internal and external mandates in various settings, individuals might evolve and change their emotioncy levels—either forward or backward—through the trans-emotioncy process (Pishghadam and Abbasnejad, 2017). This indicates that a person involved in something might get exvolved at another time or vice versa (Pishghadam, 2016). Thus, the socialization processes people experience and go through could affect their identity and emotioncy levels.

Like identity, which is continuously refined, negotiated, and influenced by different sociocultural factors (Buchanan, 2015), a person’s emotioncy level is shaped by the amount of exposure based on sensory channels to an item (Pishghadam et al., 2019). An individual emotioncy level evokes the person’s emotion kinds, shaping the person’s identity (Pishghadam, 2016). This is because a full understanding of a concept requires both emotionalization and conceptualities (Pishghadam et al., 2013a). Therefore, a person’s exposures and relationship to the world are constructed based on the context (i.e., time and space) and based on the person’s emotioncy level, whether the person is avolved, exvolved, involved, or metavolved toward a concept. The same is true with teachers who usually have to deal with various internal and external mandates in their workplace. For example, teachers who have more autonomy and agency are more likely to have higher emotioncy levels; they are more likely to be involved and metavolved in decision making and policymaking, resulting in experiencing less identity tensions or emotioncy tensions (Pishghadam, 2016). Such teachers, as a result, act as envolvers (Pishghadam et al., 2019), putting students in the center of the learning process; developing instructional materials which are based on students’ background, needs, and interests. On the other hand, teachers who constantly deal with external forces, such as institutional mandates, which require teachers to be avolved, exvolved, or involved, experience identity tensions as job-related issues are often emotionalized or de-emotionalized for them.

### Capital

The notion of capital was first introduced by Marx (1867), who mostly referred to capital as monetary practices and material possessions. Bourdieu (1986) later developed the construct by introducing other forms of capital, namely, economic, cultural, social, and symbolic capitals. This revolution paved the way for the introduction of different forms of capital, such as natural, physical, and human capital (Coleman, 1990), psychological capital (Luthans et al., 2004), emotional capital (Nowotny, 1981), and sensory capital (Pishghadam et al., 2019). The major common feature among all types of capital is having access to resources or possessing something, leading to better position and status in life. Besides, recognizing and studying the underlying social, political, and cultural factors that shape the dynamic identity language teachers have. As Varghese et al. (2005) asserted,

In order to understand language teaching and learning we need to understand teachers; and in order to understand teachers, we need to have a clearer sense of who they are: the professional, cultural, political, and individual identities which they claim or which are assigned to them (p. 22).

An individual’s involvement in something (e.g., education and crime) results in gathering capital related to the concept or item—allowing the person to become involved in something and exvolved in something else. For example, suppose a person comes from an educated family. In that case, the person is involved in education and exvolved in crime, or the person is more likely to be interested in education than crime. The achieved capital can later affect the person’s decisions, actions, and world views. For example, a teacher with higher academic qualifications is more likely to receive privilege and opportunities in the job market, or a person with a better network of social relations can gain particular benefits in one way or another. Therefore, teachers with high capitals are more likely to have a different understanding and feelings about themselves than those who lower capitals. This is because:

The structure of the field, that is, the unequal distribution of capital, is the source of the specific effects of capital, that is, the appropriation of profits and the power to impose the laws of functioning of the filed most favorable to the capital and its reproduction (Bourdieu, 1986, p. 246).

This indicates that capital (i.e., power and privilege) is influenced by how a particular field or context is structured, resulting in impacting the identity of individuals involved. Hence, there is a bilateral relationship between different types of capital and identity, meaning that one can be either the cause or the effect.

## Concluding Remarks: Integrating the New Framework Into Teacher Education

Developed based on a literature review of existing studies and theories, the new framework facilitates understanding of teacher identity development through an analytical approach. Teacher identity is a social phenomenon because its construction, negotiation, and development occur and situate within institutional sites, including teacher education programs (Varghese et al., 2005). Therefore, these settings appeared to be ideal starting points for integrating and employing the conceptual model into practice. Our proposed teacher identity development framework highlights the complexity of teacher identity and addresses various aspects of teachers’ professional development, wellbeing, classroom practices.

When developing a curriculum or syllabus, teacher educators could include scholarly articles highlighting the relationship between the six identity core components and illustrating how these constituents affect the teacher identity construction. Teachers could also be mindful of the activities they incorporate into their teaching practices, keeping the effects of the six identity constructs in mind. Such inclusion will provide space for identity negotiation and equip prospective teachers to shape a critical mentality toward their professional identity and prepare them for managing identity tensions, uncertainty, and dilemmas in the classroom and beyond while striving for a sense of continuity and coherence.

The teachers could also employ this conceptual framework to build a reflectivity toward contextual idiosyncrasies, institutional policies, their dynamic self-conception, and participatory practices within the imagined communities, and ultimately exercise their agency in the teaching profession more constructively. Our proposed framework could also be used for the pathology of teacher identity construction. Like needs analysis, the framework could be used as identity analysis in teacher education programs. It would allow them to discover different aspects of teachers’ professional identity process and open avenues for thoughts on how teachers’ identities impact their teaching practices. The practice would provide teachers with greater awareness of their multiple identities, supporting teachers to better understand themselves and recognize institutional mandates affecting their identity formation. In this respect, similar to the following questions, teacher educators could create various identity analysis questions for each of the six mentioned facets.

1. How do the mirrors of power influence teacher identity development before, during, and after the participants’ teacher education program?
2. What kinds of discourses are at play to shape teachers’ identity construction in teacher training programs? How do teacher candidates negotiate these discourses to develop their professional identity?
3. How do teachers describe their imagined and ideal classrooms? In what ways do their ideal settings differ from their real teaching contexts?
4. What kinds of investment do teacher candidates employ to exercise agency and accomplish their professional goals?
5. To what extent do teachers exercise emotioncy in their classroom practices? How does emotioncy application influence teachers’ self-efficacy?
6. What types of capital impact teachers’ professional identity?

The above six identity analysis questions could be employed as possible examples to inform teacher educators and teacher candidates of the forces that influence and shape relevant identity construction.

The framework guides the researchers to make informed decisions about the constructs they could include in their studies. It also sets the stage for a systematic and attentive investigation of teacher identity development. Moreover, it helps researchers to situate their studies within the existing literature. Therefore, the scholars could achieve a more inclusive and comprehensible understanding of teacher identity construction. However, little is known about the relationship between the core components: (a) mirrors of power, (b) discourse, (c) imagination of reality, (d) investment, (e) emotioncy, and (f) capital. Moreover, no research can be found that included the above constructs altogether. Future researchers could focus on developing a teacher identity scale considering the new framework and then examine the correlations between the constructs and variables. The framework can also open up space for researchers to investigate what changes language teacher identity bears when the teachers perform different roles as program managers/researchers across contexts.

The conceptual framework attempted to develop another perspective to examine teacher identity development, particularly for language teachers, as EFL contexts are considered fertile for studying identity (Richards, 2006). We hope this framework could support researchers as they investigate identity shifts, tensions, and emotional burdens teachers’ experiences in the classrooms.

## Data Availability Statement

The original contributions presented in the study are included in the article/supplementary material, and further inquiries can be directed to the corresponding author.

## Author Contributions

RP has developed the conceptual framework for the study, reviewed the manuscript, provided feedback, and also contributed to the manuscript revision, proofreading, and editing. Under the supervision of RP, JG, and MM wrote the first draft of the manuscript. All the authors contributed to the article and the development of the research design and approved the submitted version.

## Conflict of Interest

The authors declare that the research was conducted in the absence of any commercial or financial relationships that could be construed as a potential conflict of interest.

## Publisher’s Note

All claims expressed in this article are solely those of the authors and do not necessarily represent those of their affiliated organizations, or those of the publisher, the editors and the reviewers. Any product that may be evaluated in this article, or claim that may be made by its manufacturer, is not guaranteed or endorsed by the publisher.
